# Biomaterials for Soft Tissue Repair and Regeneration: A Focus on Italian Research in the Field

**DOI:** 10.3390/pharmaceutics13091341

**Published:** 2021-08-26

**Authors:** Maria Cristina Bonferoni, Carla Caramella, Laura Catenacci, Bice Conti, Rossella Dorati, Franca Ferrari, Ida Genta, Tiziana Modena, Sara Perteghella, Silvia Rossi, Giuseppina Sandri, Milena Sorrenti, Maria Luisa Torre, Giuseppe Tripodo

**Affiliations:** Department of Drug Sciences, University of Pavia, V.le Taramelli 12, 27100 Pavia, Italy; carla.caramella@unipv.it (C.C.); laura.catenacci@unipv.it (L.C.); rossella.dorati@unipv.it (R.D.); ida.genta@unipv.it (I.G.); tiziana.modena@unipv.it (T.M.); sara.perteghella@unipv.it (S.P.); silvia.rossi@unipv.it (S.R.); giuseppina.sandri@unipv.it (G.S.); milena.sorrenti@unipv.it (M.S.); marina.torre@unipv.it (M.L.T.); giuseppe.tripodo@unipv.it (G.T.)

**Keywords:** tissue engineering, biomaterials, silk proteins, collagen, polysaccharides, glycosaminoglycans, aliphatic polyesthers

## Abstract

Tissue repair and regeneration is an interdisciplinary field focusing on developing bioactive substitutes aimed at restoring pristine functions of damaged, diseased tissues. Biomaterials, intended as those materials compatible with living tissues after in vivo administration, play a pivotal role in this area and they have been successfully studied and developed for several years. Namely, the researches focus on improving bio-inert biomaterials that well integrate in living tissues with no or minimal tissue response, or bioactive materials that influence biological response, stimulating new tissue re-growth. This review aims to gather and introduce, in the context of Italian scientific community, cutting-edge advancements in biomaterial science applied to tissue repair and regeneration. After introducing tissue repair and regeneration, the review focuses on biodegradable and biocompatible biomaterials such as collagen, polysaccharides, silk proteins, polyesters and their derivatives, characterized by the most promising outputs in biomedical science. Attention is pointed out also to those biomaterials exerting peculiar activities, e.g., antibacterial. The regulatory frame applied to pre-clinical and early clinical studies is also outlined by distinguishing between Advanced Therapy Medicinal Products and Medical Devices.

## 1. Introduction

Regenerative medicine is the branch of medicine that aims to restore, repair or replace damaged or diseased cells, organs and tissues. It includes the generation and use of therapeutic cells, stem cells, engineered tissues and the production of artificial organs together with polymer scaffolds. Therefore, it can be defined a multidisciplinary approach that includes biology, engineering and materials science with the main goal to guarantee an adequate, functional and permanent therapy in patients with damaged organs or tissues. The approaches may include, but are not limited to, the use of soluble molecules, gene therapy, stem cell transplantation, tissue engineering and the reprogramming of cell and tissue types.

In particular, the aim of tissue engineering is the fabrication of three-dimensional (3D) scaffolds that can be used for the reconstruction and regeneration of damaged tissues. They have a crucial role because they represent an alternative to conventional implantation or replacement of organs and tissues. The scaffolds can act as acellular material, or they can be combined with cells. Another possibility is loading scaffolds with soluble molecules such as antibiotics, chemotherapeutic agents and growth factors that are transported into the surrounding environment, providing the therapeutic or regenerative effect [1].

In order to have an application in the field of tissue engineering, the scaffolds must meet some fundamental requirements, which can be summarized as follows: biocompatibility, biodegradability, processability, sterilizability, mechanical properties, porosity.

All these properties are mainly related to biomaterial properties, with the exception of porosity that refers to scaffold architecture.

**Figure 1:**
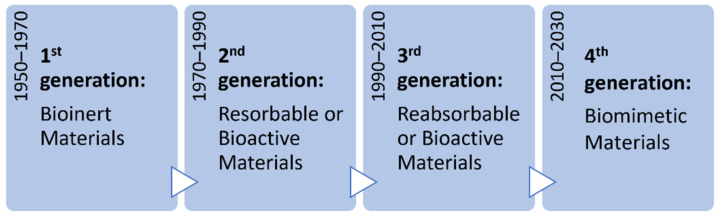
Schematic representation of scaffold categories divided in four generations.

Biocompatibility is an essential property for the biomaterials intended to be used in tissue engineering, and according to this property the biomaterials can be divided into four categories, as schematized in Figure 1.

The fundamental requirement for first generation biomaterials is to be bioinert, therefore, to possess physical properties equal to those of the replaced tissue and minimum toxicity.

The second generation is characterized by materials that are either resorbable or bioactive. A resorbable biomaterial degrades chemically and is reabsorbed without leaving a trace, so that it is replaced by the tissue that hosts it; a biomaterial is defined bioactive when it is able to cause controlled actions and reactions in the physiological environment. Third generation materials are both resorbable and bioactive. Biomimetic materials represent the fourth generation of materials; they are immunologically inert and able not only to replace the original tissue, but also to exchange signals with the host cells.

Biodegradability is defined in the literature as the capability of being degraded by biological activity [2]. Degradation is a process that involves breaks of the polymer chains and modifications of its chemical structure. It is usually subdivided into four phases: water uptake, loss of mechanical properties, reduction of molecular weight and mass loss. The degradation time of the materials constituting a scaffold must be closely coordinated with that of new tissue regrowth. In fact, too rapid degradation of the scaffold matrix does not allow a complete and strong tissue to form; on the contrary, too long degradation times induce formation of fibrous tissue around the scaffold in an imperfect or incomplete way avoiding regeneration process.

Processability is the ability of a biomaterial to be easily workable so as to assume the desired morphological and dimensional characteristics through a reproducible manufacturing process. Materials with a softening temperature compatible with the process temperature, with mechanical properties giving the scaffold the desired strength for its intended use, which is sized depending on the tissue to be regenerated, with sufficient permeability for the passage of oxygen and nutrients and with a suitable degradation rate are therefore preferable.

Sterility is a compulsory requirement for each device to be implanted in the human body. The material must be sterilizable and to resist to sterilization processes without degrading. If the material does not resist to common terminal sterilization methods, an aseptic protocol should be set up in order to manufacture the scaffold in sterile conditions. (EMA, 2019, Guideline on the sterilization of the medicinal product, active substance, excipient and primary container). Sterilization is an essential step as scaffolds could be contaminated by a wide range of microorganisms.

A scaffold should have similar mechanical strength in order to temporarily replace those characteristics the tissue is lacking, and it should have sufficient strength and mechanical stability to withstand its implant and the typical loading conditions of in vivo tissue stresses. The mechanical properties of a material are related to loading and deformation. At the basis of this definition, there are two concepts: firstly, when a material undergoes loading, deformation occurs; secondly, the relationship between loading and deformation defines material stiffness. The literature on mechanical properties of biological tissues has been recently reviewed by Guimaraes and collaborators [3]. Moreover, some experimental works discuss in details scaffolds mechanical characterization [4,5].

The scaffolds must be highly porous, with pores of suitable size in order to allow cell colonization, i.e., their penetration, adhesion and proliferation. In addition, porosity must be sufficient to allow diffusion of nutrients and elimination of waste. An optimal scaffold model should promote cell proliferation and production of a cell specific matrix, which should replace the supporting role of the scaffold after its degradation.

Due to the importance of biomaterials in tissue engineering and regeneration, and to the wide number of biomaterials studied in the recent years, a lot of experimental papers and reviews can be found in the literature on this topic. In this International scientific context, the present special issue aims to gather and discuss the most recent studies performed by Italian scientists. Therefore, the review focuses on Italian research in the field of tissue engineering, in order to fit the aim of this special issue. The reviewed experimental researches involve both natural and synthetic polymers, and main focus is on soft tissues regeneration, i.e., cartilage, ligament and tendon, skin, neurons, vasculature, esophagus. The manuscript is divided in sections, each one dedicated to a class of biomaterials, namely: silk proteins, collagen, polysaccharides glycosaminoglycans (GAGs) and biodegradable polyesters. Each section reports a description of the biomaterial, or biomaterial class, focusing on biomaterials properties and formulations (i.e., sponges, hydrogels, mats and fibers, films, nanoparticles) studied for tissue regeneration purposes. The different applications to tissue repair and regeneration are always indicated and briefly discussed in the text for all biomaterials reviewed. Final chapters with future perspectives of the discussed biomaterials and regulatory aspects related to medicinal products and medical devices intended for tissue engineering are also reported.

## 2. Silk Proteins

Silk is a natural material produced by many arthropods, such as silkworms and spiders [6]. Silk proteins used in the biomedical field are principally extracted by *Bombyx mori* cocoons composed of two proteins, silk fibroin (SF) and silk sericin (SS), characterized by different structures and properties. Silk fibroin is a fibrous and hydrophobic protein that represents about 65–85% *w/w* of cocoon and is widely used in the textile industry for thousands of years. During the last two decades, SF was studied mainly for biomedical applications and it was approved by the Food and Drug Administration (FDA) as a suture thread (Surusil^®^, Suru; Sofsilk™, Covidien) and as a scaffold (Seri^®^ Surgical Scaffold, Allergan, Medford, MA, USA) [7,8]. Silk sericin is a globular hydrophilic protein that covers the SF filaments and maintains the structural integrity of cocoon. It was routinely discarded by the textile industry through a degumming process; however, during the last decades, it has been demonstrated that SS is a bioactive compound with antioxidant, anti-tyrosinase, anti-elastase and anti-bacterial properties [9,10], other than showing anticoagulant, anticarcinogenic characteristics, it is biocompatible, UV resistant and able to absorb moisture [11,12,13].

The biological and mechanical properties of SF and SS make them ideal candidates to produce scaffolds and nanostructured drug delivery systems for tissue repair and regeneration. A crucial step for use of SF and SS is the complete separation of these two proteins; in fact, it was demonstrated that SF and SS’s combination was associated with adverse immune response [6]. Table 1 reports the principal properties of SF and SS in systems for reparation and regeneration

### 2.1. Silk Fibroin

Silk fibroin is the main component of *B. mori* cocoon and consists of a heavy chain (H) and a light chain (L) with a molecular weight of 390 and 26 kDa, respectively [31]. In cocoon structure, H and L chains are linked by disulphide bonds to make a complex which is linked to a glycoprotein of 25 kDa (P25) at 6:6:1 (H:L:P25) molecular ratio [32]. The abundance of Glycine and Alanine residues of H-chain concur to stabilize the typical antiparallel β-sheet crystalline form of SF. Silk fibroin exists in two principal conformations with different solubility and stability; silk I is the metastable form of SF and includes bound water. Silk II conformation is characterized by a high stability, rigidity and tensile strength [33]. The use of native SF allowed obtaining a stable scaffold with predominant Silk II protein conformation; on the other side, using soluble SF, we can obtain regenerated SF-based scaffolds that need to be subjected to specific treatments for complete conformational change from Silk I to Silk II [31].

Silk fibroin can be considered a unique material thanks to its peculiar structure and characteristics such as tunable mechanical properties, low immunogenicity, biocompatibility and biodegradability. The mechanical properties of SF-based materials can be changed by using native or regenerated SF (RSF) and selecting other compounds that allow obtaining composite scaffolds [34,35]. Silk fibroin was considered an inert material inducing negligible in vivo reaction; low immunogenicity and biocompatibility of SF were demonstrated for many years, both in vitro and in vivo [36]. Its degradation rate is correlated to the β-sheet content and, for this reason, it is faster in regenerated SF with respect to native SF. Overall, non-crystalline regions of SF can be degraded by some enzymes without the formation of toxic by-products [14].

Concerning the application field, the tunable properties of SF allowed producing scaffolds with different morphologies and physical-chemical characteristics. Therefore, during the last two decades, SF was used to obtain different scaffold structures for application in cartilage, ligaments, skin tissue regeneration and wound healing: sponges, hydrogels, films, mats and fibers or 3Dprinted scaffolds. Moreover, also SF nanoparticles were proposed for tissue repair and regeneration.

#### 2.1.1. Sponges

Porous sponges can be prepared using different approaches such as freeze-drying [37], gas foaming [38], freeze-drying/foaming [39] and porogens [40]. The pore size of the scaffold structure can be modulated, changing the production process parameters, such as RSF solution concentration and pH, freezing temperature and rate, porogen type.

The most used porogens are salts and sugars with homogeneous size distribution; the mean size of porogens directly affect the scaffold pore size. Considering the freeze-drying technique, a slow freezing rate induce the formation of larger pores, while a microporous scaffold can be obtained, reducing the large ice crystal formation by the improvement of freezing speed.

Overall, SF sponges were widely studied for tissue engineering applications thanks to their favourable porous interconnection and to the ability of modulating the pore size. The mechanical strength of SF sponges allowed to use them as a bone substitute; furthermore, the combined use of SF with hydroxyapatite and/or collagen, the main components of human bones, allowed extracellular matrix deposition and promoted the osteogenic differentiation of human mesenchymal stem cells [41]. In vitro experiments also demonstrated that SF sponges are optimal supports for chondrocyte proliferation and functionality, paving the way to use these scaffolds for cartilage, ligament and tendon tissue engineering.

#### 2.1.2. Hydrogels

Silk fibroin hydrogels are hydrophilic networks that can absorb high water amount, maintaining their 3D structure. Gelation techniques, including chemical and physical methods, were based on the transition of RSF from Silk I (sol) to Silk II (gel) conformation. Tunable characteristics of SF hydrogel permitted to optimize tissue regeneration process by the controlled release of cells, growth factors and bioactive molecules.

Physical methods based on the application of physical stimuli affecting the SF hydration, folded state and aggregation can be found in the literature. The main studied techniques include temperature variation [42,43], shear force application [44], such as vortex mixing [44], ultrasound [45] and electric fields [44,46,47].

Chemical methods are more numerous and include precipitating agents, pH variation, high-pressure CO_2_, chemical stabilization and chemical modification of RSF. Precipitating agents include salts whose ions are capable of inducing deprivation (salting out) or addiction (salting in) of solvent to the protein: modification of SF hydration effectively causes protein precipitation and hydrogel formation [46]. Polymers, such as polyethylene glycols and polyethylene oxide (PEO), can be used as precipitating agents to promote protein-protein aggregation. Organic solvents can dehydrate α-helices structures of SF, encouraging the formation of β-sheet regions and consequent protein precipitation. The pH of solubilized proteins is directly correlated to their surface charge and their aggregation state; protein precipitation could be induced working at solution pH close to the protein isoelectric point (pI). In fact, the protein charge-charge repulsion is minimal when solution pH is equal to pI value. A similar effect can be obtained by adding high-pressure CO_2_, a volatile acid, to the SF solution; this approach allows pH modification and final efficient recovery of the solvent circumventing solvent removal steps [48]. Stable SF hydrogels were also obtained using a chemical crosslinker (e.g., hydrogen peroxide and horseradish peroxidase) which formed covalent bonds with protein chains.

Hydrogels allow both cell encapsulation in the hydrophilic matrix and cell loading on scaffold surface. Overall, the possibility of making injectable scaffolds and modulating the hydrogel properties in terms of shape, porosity, and stability encouraged the use of these scaffolds for both hard and soft tissue engineering. Encouraging results were also obtained when SF hydrogels were used for cartilage and nucleus pulposus regeneration [46,49]. Regarding soft tissues, good results were obtained in both neuronal and vascular regeneration.

#### 2.1.3. Films

Silk fibroin films can be easily obtained by solution casting methods; overall, the most used approach involved RSF films being casted in a selected mold and then dried or freeze-dried. A drying process at constant temperature and humidity allowed to produce a more homogeneous structure avoiding “valley and ridges” formation. As previously underlined, RSF can be combined with other biomaterials and polymers (e.g., PEO, collagen, pectin and glycerol) to optimize the scaffold properties according to the field of application [50,51]. The bidimensional structure of SF-based films makes them an ideal candidate for skin regeneration. Recent research demonstrated the efficacy of SF films in murine and porcine wound models; developed scaffolds significantly reduced the wound healing time and improved skin regeneration with respect to commercial dressings [52]. These results were confirmed by a clinical trial with 71 patients, which also demonstrated a significant reduction of adverse events compared to a commercially available wound dressing [52]. Preclinical and clinical evidence are paving the way for clinical use of SF films in skin repair and regeneration.

#### 2.1.4. Matrices and Fibers

In contrast with previously presented scaffolds, matrices (mats) and fibers can be obtained using both regenerated and native SF. These scaffold types are proposed to be used alone as cell supports or in combination with micro and nanoparticles loaded with bioactive molecules able to improve tissue regeneration and repair.

Native fibroin-based non-woven mats were prepared by the water-entanglement technique; the scaffolds resulted as ideal supports for cell adhesion thanks to their high surface area, high porosity, rough morphology and similarity to extracellular matrix [53].

The most commonly used techniques for regenerated fibroin-based mats and fibers were electrospinning, wet-spinning and dry-spinning. Electrospinning allowed to produce nanometer scale fibers starting from a polymeric solution by an electrohydrodynamic and voltage-driven process [22]. The SF solution was pushed through a metal needle, controlling flow rate and subjected to high voltage (1–30 kV). This mechanical stress induced a conformational change of SF to Silk II structure, promoting fiber formation [54]. Wet and dry-spinning techniques allowed to obtain micrometric fibers and can be used in combination with other biopolymers [33].

Mats and fibers were mainly proposed for skin regeneration and wound tissue healing. SF mats loaded with mesenchymal stem cells allowed subcutaneous compartment’s vascularization, which vascular network is an essential condition for the nutrient supply of all cells involved in the tissue regeneration process [55,56].

#### 2.1.5. 3D Printed Scaffolds

3D printing technologies, and in particular bioprinting technique, were recently proposed to improve the definition of SF scaffolds. A preliminary Computer Aided Design (CAD) allowed to obtain a macroscopic structure that could be regulated into meso and nanostructures applying mechanical stresses and dopants [33]. 3D printing is considered a promising technique and a future profitable alternative to diverse current clinical treatments. The success of 3D bioprinted products is strictly correlated to bioink choice that should be performed, together with the cells and bioactive molecules to be loaded, already in the early stage of 3D bioprinting procedure. Important and unavoidable bioinks requirements are their compatibility with both cell culture and the selected printing process [25]. During the last decade, many researchers explored the feasibility to use SF as an effective bioink. Overall, published data demonstrated that pure fibroin resulted in a non-optimal bioink; hybrid bioinks could improve the printability, in term of rheology and viscosity, of silk fibroin-based solutions. Gelatin, collagen, chitosan, alginate and polyethylene glycol were combined with SF and used as bioinks in scaffold development for bone, cartilage and vascular tissue engineering.

#### 2.1.6. Nanoparticles

Silk fibroin nanoparticles can be added to 3D scaffolds to improve tissue regenerative potential of the final product. Nanoparticulate drug delivery systems allowed to control mechanical properties of scaffolds and they are also widely used to control release of bioactive agents. Silk fibroin nanoparticles can be prepared using different techniques such as salting out, crosslinking reaction, reverse microemulsion, polymer blending and desolvation. The last method was one of the most used because it allowed obtaining pure SF nanoparticles with a mean diameter of about 100 nm. Silk fibroin nanoparticles improved the bioavailability of hydrophobic drugs and reduced the toxicity of many bioactive agents [57,58,59]. Furthermore, SF nanoparticles could be functionalized to obtain smart drug delivery systems; this approach allowed to obtain site-specific delivery of anticancer drugs, boosting activity and sparing healthy organs [60]. Addition of SF nanoparticles to 3D scaffolds represents a good strategy to obtain more efficient systems for tissue engineering applications.

### 2.2. Silk Sericin

Silk sericin (SS) contributes to the maintenance of the structural integrity of *Bombyx mori* cocoon, acting as a binder component between SF fibers. Silk sericin is a glue-like protein with an amorphous structure characterized by partial water solubility. Serine, aspartic acid, glycine and threonine are the most abundant amino acids of SS, overall including about 70% hydrophilic amino acids [61]. The secondary structure of SS is mainly organized in random coil and β-sheet, and it is possible to observe a sol-gel transition due to the transformation of random coil structure to β-sheet by temperature lowering. The gelation process is reversible, and the β-sheet structure easily degrades by high-temperature treatment [10]. Silk sericin has broad molecular weight (from 10 to 400 kDa), changing depending on the extraction method, pH and temperature [62]; those peptides with low molecular weight (about 20 kDa) were mostly studied for cosmetic applications while higher molecular SS weight was mainly investigated as biomaterial and for development of drug delivery systems [8,9,63,64,65,66].

SS-based scaffold prepared solely with this protein show some issues such as fast degradation rate, high release rate of the loaded active molecules and poor mechanical characteristics [10,67]. To overcome these limitations, principally correlated to the hydrophilic profile of SS, some researchers proposed to add other polymers or to modify the protein structure by physical and chemical treatments. These approaches allowed to obtain sponges, hydrogels, films and nanoparticles for tissue engineering applications.

#### 2.2.1. Sponges

Silk sericin-based sponges were principally prepared by lyophilization: chitosan and carboxymethyl cellulose were largely proposed as blended polymers. The porous structure of these scaffolds allowed to support fibroblast and keratinocyte proliferation. Moreover, addition of antimicrobial agents resulted in an optimal strategy to minimize bacterial infections. Silk sericin could be used as a structural component of these scaffolds and as a bioactive compound in such a way the antioxidant and antibacterial activity of SS supports and improves tissue repair and regeneration.

Silk sericin-based sponges were principally proposed for skin lesion and ulcer treatment and nerve guidance to promote nerve regeneration.

#### 2.2.2. Hydrogels

Hydrogels were studied mainly in tissue engineering field because they are injectable and allowed to obtain a minimal-invasive treatment. A pure SS hydrogel was obtained using glutaraldehyde as crosslinker agent [28], whereas in situ forming hydrogels were prepared by chemical modification of the protein with methacryloyl or polyacrylamide [68]. A photoluminescent hydrogel was prepared by using calcium chloride or glutaraldehyde as alginate and sericin crosslinker [69]. A recent study proposed 3D printing technology to develop a hydrogel composed of silk sericin and methacrylic-anhydride-modified gelatin [70]. This scaffold presented optimal characteristics as wound dressing: high swelling degree, regularly microporous structure, controllable degradability, good biocompatibility and high transparency. Using this scaffold type it could be possible wound visual inspection without removing the scaffold and along tissue regeneration process.

Overall SS-based hydrogels enhanced cell adhesion, proliferation and long-term survival and were proposed for bone, cartilage, nerve and skin regeneration.

#### 2.2.3. Films

Silk sericin was blended with other polymers to obtain films for wound healing applications. Sericin/collagen and sericin/chitosan films were crosslinked with glutaraldehyde to improve the tensile strength with respect to the un-crosslinked membrane [71]. Ethanol treatment was proposed to gel a sericin solution containing bacterial cellulose as an antibacterial agent [72]. Cellulose nanofibrils were combined with silk sericin to obtain nanocomposite films prepared with an eco-friendly ultrasonication process [73]; the addition of nanofibrils enhanced tensile properties and hydrophilic capacity of the developed films.

#### 2.2.4. Nanoparticles

As previously described, the physical-chemical properties of silk sericin limit the possibility to obtain pure sericin nanoparticles. Sericin and poloxamer were used to produce nanoparticles with a micellar structure characterized by a hydrophobic core and a hydrophilic corona [74]; this amphiphilic structure allowed to load bioactive molecules with different solubility properties. Sericin-based nano-drug delivery systems addition to 3D scaffolds could improve the biological properties of final constructs. As an example, the antibacterial potential of SS could be exploited to reduce infection risk during wound healing process.

## 3. Collagen

Collagen (CL) is one of the most represented proteins of the extracellular matrix (ECM), together with other proteins such as elastin and fibronectin [75]. Collagen participates in the 3D structure that surrounds cells and guarantees the maintenance of tissue’s mechanical properties. The organization of cell growth is regulated through cell-collagen interactions based on recognition of collagen cues by different kinds of cell surface proteins. Collagen is quite resistant to degradation from proteolytic enzymes, although it is a substrate of metalloproteinases, that act on collagen in continuous re-modeling of ECM [76].

Collagen functions are supported by the peculiar structure of the protein. 29 types have been described, all of them organized in a triple helix, but only 5 have the property to form a quaternary structure that leads to organization into fibers. Among these is CL type I, the most abundant in nature and the most usually employed as a biomaterial for tissue repair. It is obtained especially from cows, pigs and sheep, thanks to the high homology with the human protein. Quite recently, alternatives are however offered by recombinant protein and by the even more promising extraction from marine organisms such as fish, jellyfish and sponges [77].

The ability to self-organize in fibers makes collagen a natural support, that inspired CL-based formulations since first development of scaffolds for tissue regeneration [76,78]. Many devices based on CL were developed for skin healing, to be applied in case of wounds and burns, and a certain number reached commercialization and clinical use. Considering the kind of structure, most of them are classified as pads, but also membranes, gels, powders, sponges and compressed sponges, acellular scaffolds and cellular matrices cultivated with skin cells can be found [79]. Other possible applications of CL involve regeneration of cornea, whose stroma is well known to be mainly made of CL, especially employing CL gels. Compressed gels were proposed to improve CL concentration and structure rigidity [80,81]. Collagen-based scaffolds were proposed also in the case of bone regeneration, although in this case mimicking ECM is especially challenging due to the complexity of the natural arrangement of the collagen fibers. Some promising results have been achieved with freeze-dried CL scaffolds. In this case a critical parameter seems to be pore size, which should be relatively high, about 100–800 µm, to obtain the best results [80]. The coating of implants with type I collagen demonstrated to be a successful strategy for the guided tissue regeneration in the case of periodontal applications [82]. Among the authors that selected CL to design support systems for the reparation of tendons, Gigante et al. [83] prepared membranes by deposition of subsequent layers of a CL gel on molds that were electrostatically charged. In this way a multilamellar structure was obtained, relevant for fastening cell growth of human fibroblasts and human tenocytes [83].

The mechanical properties on which CL functions rely strongly depend on natural crosslinking that stabilizes the native CL structure [84]. However, this is generally lost during the extraction processes, leading to less favorable mechanical properties and stability. Therefore, addition of exogenous crosslinking is a relevant aspect to obtain materials with adequate properties for tissue engineering and reparation. The most common methods are usually classified as chemical, physical and enzymatic crosslinking, as well described by a recent specific review of Gu et al. [85]. Physical treatments usually result in lower improvement of mechanical properties and protection towards degradation, whereas in the case of chemical reactions, attention must be paid to the residuals of chemical agents and therefore to safety concerns. For example, a well-known question is related to the use of aldehydes. In all cases, the effect of crosslinking on the ability of CL to cell attachment properties must be verified, as the cell-binding domains can be less exposed after the crosslinking step [86].

A group of the University of Salento performed a study on crosslinking obtained by dehydrothermal treatment (DHT) at different temperatures, and the relevance of the procedure on denaturation extent [87]. They also compared DHT with Ethyl-3-(3-dimethylaminopropyl)carbodiimide)-based (EDC) crosslinking. In this work they highlighted that the triple helix unfolding that follows DHT treatment, improves cell recognition by increasing the exposure of the RGD (Arg-Gly-Asp) ligands, an effect that was not observed with EDC crosslinking [88].

The same group, in a more recent work, compared different crosslinking methods for type I CL. The comparison was based on both elastically effective crosslinking (ρxel), which is related to the intermolecular crosslinks, and chemically effective crosslinking (ρxch), which also considers the intramolecular ones. They compared some chemical methods, based on the use, as crosslinking agents, of two aldehydes (glutaraldehyde or formaldehyde), genipin, dimethyl suberimidate or carbodiimide. In all cases, these performed a physical dehydrothermal crosslinking by heating the freeze-dried CL under vacuum at 120 °C for 72 h. The ranking of the effect of the different chemical agents on ρxch was not superimposable to that of the same chemical agents on ρxel. Carbodiimide crosslinking promoted a higher amount of intramolecular bonds than other treatments, such as the one involving aldehydes. The relevance of ρxch and ρxex on the physical and mechanical properties was considered by the authors. Denaturation temperature (Td), was correlated to intramolecular crosslinking, whereas no correlation was evidenced between either ρxch or ρxel and degradation temperature. Collagenase degradation was especially sensitive to ρxel, making dehydrothermal CL degradation faster (1 h) than that observed for carbodiimide (4 h), while aldehyde treated samples showed negligible weight loss. A correlation was found between water uptake and ρxel and the same was observed for in vitro half-life of crosslinked collagen scaffolds [86].

Montalbano et al. [89] developed a CL construct by extrusion 3D printing technology. The type I CL was associated with nanosized mesoporous bioactive glasses (MBG) loaded with strontium, added for its positive effect on bone formation. To improve CL mechanical properties, a water-ethanol solution of genipin was chosen as a crosslinking agent, thus obtaining an improvement of the viscoelastic properties and a better support to cell growth. The crosslinked system stability was confirmed by a reduction of enzymatic and hydrolytic degradation rate [89].

An alternative approach to improve CL mechanical properties and stability involves combining the protein with different polymers or materials. Literature reports studies that involve polymers such as silk fibroin [90], alginate and fibrin [91], hyaluronic acid and chitosan [92].

In Caddeo et al. [93], a newly synthesized polyurethane (PUR) was combined with type I CL and associated with a glass-ceramic surface in order to more closely reproduce bone extracellular matrix for bone tissue engineering. The hydrophilic and well-tolerated polyethylene glycol, the non-toxic 1,6-hexamethylene diisocyanate and N-BOC-serinol were selected by the authors to obtain a water-soluble polyurethane that could easily be co-solubilized with CL and endowed with free amino groups useful to its interaction with a bioactive glass exposing amino groups thanks to a previous silanization process. The mixture of polyurethane and collagen was linked to ceramic glass thanks to genipin [93].

Inorganic materials are also, often studied in association with CL. Colaço et al. [94] studied the interaction of CL with hydroxyapatite nanoparticles of different size (from 30 to 130 nm) and shape (rods and platelets) to better understand their interaction with the protein. The small rod shaped hydroxyapatite nanoparticles decorated collagen fibers gave, in turn, large self-assembling fibrillar structures, characterized by negative charges that could be exploited in layer-by-layer assembly, tuning the supramolecular structure for the requirements of different biomedical applications [94]. The combination of CL and hydroxyapatite was studied also by Yu et al. [95], which compared different microstructures, cellular or lamellar, to improve scaffold efficiency in bone regeneration [95]. Debons et al. [96] studied the association of CL with silica nanoparticles, largely considered together with CL for the regeneration of dermis, bones and nervous structures. In particular, the authors evaluated the effect of this combination to obtain filaments of collagen and silica nanoparticles on PC12 cell lines as a model of neuronal differentiation in vitro [96].

Some of the most commonly explored combinations involve extensively used biodegradable polymers such as polylactide (PLA), polylactide-co-glycolide (PLGA) or poly-ε-caprolactone (PCL). These polymers present the advantage of good mechanical resistance and of possible modulation of the degradation times, but a disadvantage is that they are quite hydrophobic. The presence of CL in mixture with them improved the material wettability, and the positive interaction with the biological substrates, due also to the maintenance of CL recognition sites for cell adhesion [97]. The literature reports different studies on this subject, demonstrating the still recent interest towards this aspect.

Among the most recent papers that involve PCL and collagen combinations, Oh et al. used 3D printing to combine PCL and fish collagen in scaffold for bone regeneration [98].

Some papers address the combination of PCL and CL in electrospun fibers preparation. Dulnik et al. quite recently described the preparation and the biodegradation of CL electrospun fibers in different solvents and the effect of these conditions on cell recognition and growth [99,100].

In Miele et al. [97], soluble CL was electrospun together with PCL in 90% acetic acid at different PCL and CL ratios adjusting the process parameters thanks to the results of a design of experiments. In the dry state, the addition of CL up to the 1:1 weight ratio in the formulation drastically decreased elongation and increased the Young’s modulus and a more fragile material, with poor deformability, was obtained. In the wet state, anyway, for CL/PCL 1:1 (*w/w*) membranes, the combination of the highest content of CL with the more plastic PCL led to nanofibers with the highest capability of deformation. After wetting, good morphological stability was observed for all the samples up to one week, although the release of the soluble, not cross-linked CL was complete in this same period. The systems however after one week still supported the growth of fibroblasts to an extent dependent on CL concentration and more efficiently than analogous systems based on gelatin. The PCL-CL blend seemed in this case an effective combination of PCL capability to support the cell growth and CL improvement of wettability and cell growth stimulation despite the solubilization in the fiber microenvironment. In this case, the co-electrospun system can be seen as a PCL scaffold loaded with CL, and slowly releasing it, to exploit its compatibility and cell stimulation activity [97].

In Gouveia et al. [101], CL represents the core of fibers with a more complex structure, aimed at the repair of the anterior cruciate ligament. The inner core was made of type I freeze-dried CL, capable to exploit the positive interaction with cells, whereas the outer shell was made of electrospun fibers of PCL, which guarantee to the system the necessary mechanical properties. Peculiar advantages in the core properties come from the doping of collagen with proteoglycans and glycosaminoglycans, especially chondroitin sulfate [101].

Another quite widely explored combination involves PLA/PLGA polymers. Qiao et al. mixed PLA and CL in different ratios, finding that the 60/40% *w/w* combination allowed to obtain a scaffold with good stability, although further stabilization by glutaraldehyde crosslinking was required to support cell cultures up to five weeks culture. Despite the partial degradation observed during the electrospinning process, CL maintained its ability to promote attachment and growth of human bone marrow stromal cells [102].

PLGA-CL hybrid meshes have been proposed, made of PLGA knitted meshes combined with CL sponges. The collagen was added to fill the meshes of PLGA, freeze-dried and subsequently cross-linked with carbodiimide and N-hydroxysuccinimide. The hybrid systems have proved to be suitable as support for the growth of human mesenchymal cells [103].

A different approach was used by Bellini et al. [104], who synthesized a new CL material derivatized by grafting PLA chains. The synthesis was performed through a heterogeneous phase reaction involving PLA carboxyl groups and the amine and hydroxyl groups of a CL sponge. As a result, PLA was grafted along CL chains thanks to amide and ester functions. The new material was characterized by low water uptake and good mechanical properties, suitable for use as a scaffold for tendon regeneration. Enzymatic degradation studies showed improved resistance and the biocompatibility was comparable to that of native CL as for cell viability and proliferation assays [104]. Membranes based on this material were evaluated in vitro and in vivo on a rat model presenting acute lesion of Achilles’ tendons to verify biocompatibility. The ability to support tendon regeneration and to prevent postsurgical adhesion was confirmed [105].

Salvatore et al. [106] associated CL to a different biodegradable polymer, the poly(3-hydroxybutyrate) (PHB), a polyester obtained by bacteria and algae, characterized by good biocompatibility. Similar to PLGA or PCL, PHB is however quite hydrophobic and does not present cell-binding sites. The authors processed it in an electrospinning process at three different ratios with collagen ranging from zero up to 50%. The increase in collagen percentage controlled morphological and mechanical properties of the fibers, resulting in larger fibers but higher thermal decomposition temperature and faster degradation in the aqueous environment. Fibroblasts however showed good proliferation on these membranes for at least 6 days of incubation [106]. Some of the principal properties of collagen in repair and regeneration are schematized in Table 2.

Most of the properties of the collagen-based systems, such as biocompatibility and biodegradability derive from the natural origin and chemical structure of the material, and are more affected by modifications such as cross-linking than by the scaffold type. Similarly, the mechanical properties in hydrated conditions such as those encountered after application to target tissues, are also dependent on fast collagen dissolution [97], unless one of the strategies previously described and schematically recalled in Table 2 are put in action. The crosslinking strategy seems the principal factor responsible for reaching high elastic modulus values, with different results depending more on the kind of crosslinking process than on the scaffold type [80]. This is especially true in the case of fibers and films, for which it is reported that chemical crosslinking leads to the highest elastic modulus values, in the MPa order, while physical crosslinking usually leads to relatively lower resistance [80,84]. Grafting or mixing with other materials or polymers further modulates degradation rate or mechanical properties for all the scaffold types proposed. Considering the chemical nature of the material, sterilization always relies on gamma irradiation or ethylene oxide exposure.

## 4. Polysaccharides

In the past few decades, significant attention has been paid to the use of polysaccharides as adequate materials for tissue engineering and regenerative medicine due to their biocompatibility and structural similarity to the extracellular matrix components. Abundant availability and unique biological activity of these natural polymers make them and/or their semi-synthetic derivatives suitable candidates for the development of novel formulations resembling the natural structure and functionality of damaged tissues.

Among various polysaccharides, chitosan and its derivatives, alginate, gellan and glycosaminoglycans, in particular hyaluronic acid and chondroitin sulfate, represent attractive candidates for tissue regeneration.

### 4.1. Chitosan and Chitosan Derivatives

Chitosan (CHS) is a linear natural carbohydrate biopolymer derived from chitin with structural similarity to the glycosaminoglycans of ECM involved in cell-cell adhesion [109]. The hydrophilic structure of CHS promotes cell adhesion, proliferation and differentiation of different types of cells, and its polycationic nature at a mildly acidic condition allows immobilization of negatively charged enzymes, proteins and DNA for gene delivery [110,111].

In addition to being employed for the development of drug delivery systems [112], CHS and its derivatives have been fruitfully used in formulations as biopolymers intended for repair and regeneration of various damaged soft tissues such as skin lesions (chronic ulcers, and burns), mucosal damages, cartilage, bone, nerve and tendon injuries [113,114,115,116].

In this paragraph, the attention will be focused on the development of CHS-based formulations for wound healing and mucosal application.

The polycationic character of CHS determines its unique bioactive properties, such as hemostatic mucoadhesive, proliferation and antimicrobial ones, which make CHS a multifunctional polymer for wound healing [117,118,119]. Moreover, thanks to its well-known biocompatibility, non-toxicity and biodegradability, CHS use in wound dressings is approved by FDA and several CHS containing hemostatic products are currently available on the market in the United States [120].

Due to the remarkable properties above mentioned, CHS and its derivatives have been widely exploited for the preparation of a variety of innovative drug delivery systems intended for the treatment of chronic skin ulcers, including either 3D (hydrogels, sponge-like dressings) and 2D (films and membranes) scaffolds [117,121].

Chitosan alone or in complex with other natural polymers has also been used as component of asymmetric membranes, usually making the underlying layer in contact with the damaged skin. Glycosaminoglycans, especially HA, are those polymers most fruitfully employed in combination with CHS [122,123]. Examples of CHS-GAGs based systems are reported in the literature, such as a human keratin-CHS membrane [124] and a CHS-chondroitin sulfate polyelectrolyte [125] complex that were described to possess mechanical properties, antimicrobial effect and cytocompatibility suitable for wound healing applications. Moreover, medications made from CHS can be loaded with growth factors (GFs) and cytokines or hemoderivatives, such as platelet rich plasma (PRP) or platelet lysate (PL), rich in GFs, in order to improve their performance in wound healing process. Growth factors, in fact, play a pivotal role in all stages of wound healing, modulating cell proliferation, migration and differentiation.

At the University of Pavia, Department of Drug Sciences, a many years’ experience has been gained in the development and characterization of wound dressings based on CHS and loaded with PL. Sponge-like dressings intended for the treatment of chronic skin ulcers were obtained by freeze-drying blends of PL with either chitosan glutamate (CHSG) or sodium hyaluronate [126]. The formulations contained glycine (GLY) as cryoprotectant agent and water as plasticizer and were loaded with different amounts of PL. Depending on their composition, the obtained sponge-like dressings showed different mechanical and hydration properties, tailored for the treatment of wounds characterized by different exudate amounts. In particular, when placed in contact with phosphate buffer pH 7.2 (medium simulating wound exudate), HA-based dressing immediately gelified and dissolved a few minutes, whereas CHSG-based formulations maintained their structure up to 6 days. When glycerophosphate (GP) was added to CHSG based dressings, they underwent complete gelation after 24 h. The sponge-like lyophilized dressings containing PL, when subjected to a proliferation test on human fibroblast cells, showed percentage of cell proliferation values comparable to those obtained with fresh PL, indicating that the freeze-drying process and the excipients employed did not hamper PL GFs activity. In a subsequent work, sponge-like dressings based on CHSG (high molecular weight), glycine and SS intended for the treatment of chronic skin ulcers were developed. The dressing design and development was assisted by a Design of Experiment (DoE) approach. The optimized formulation was characterized by optimal mechanical properties, cell proliferation enhancement and antioxidant activity on human fibroblasts [127]. The CHSG/glycine/SS dressing was loaded with PL following two different approaches, and tested in vitro on fibroblast proliferation in order to evaluate the synergic effect of SS on cell proliferation. The formulations demonstrated to increase in vitro the number, not only of viable fibroblasts, but also of those in the proliferative phase. Moreover, histological evaluation of human skin strips placed in contact with the PL-loaded dressings indicated their positive effect on dermal matrix reconstruction [128].

More recently, a powder formulation for the delivery of Manuka Honey (MH) bioactive components and PL in chronic skin ulcers was developed [129]. It was made from pectin (PEC)/CHS particles prepared by ionotropic gelation in the presence of CaCl_2_. The powder development study involved investigation and set up of the formulation composition (i.e., CHS and calcium chloride concentrations) and of the preparation process conditions (i.e., cure time in the cationic solution). Two different fractions of MH were also considered: Fr1, rich in methylglyoxal and Fr2, rich in polyphenols, and Fr1 proved to be the one able to enhance in vitro proliferation of human fibroblasts. In vivo efficacy of PL- and Fr1-loaded particles was assessed on a rat wound model. Both treatments markedly increased wound healing to the same extent after 18 days (remaining wound area about 30% *versus* about 60% of control).

The positive results obtained by encapsulating silver sulfadiazine (AgSD) in polymeric micelles based on CHS oleate salt are an example of the usefulness of CHS derivatives in wound healing treatments [130]. This recently developed amphiphilic derivative of CHS was proposed to improve aqueous dispersion of poorly soluble drugs such as clarithromycin, overall anti-infectives used in wound healing [131] and antioxidant agents like alpha-tocopherol [132]. Both chitosan and oleic acid are described in the literature for their antimicrobial activity, that is maintained in CHS oleate salt and can support its efficacy towards both bacterial and fungal strains of lipidic phases such as essential oils [133]. Chitosan oleate demonstrated good compatibility with PL and improved GFs release as confirmed by Platelet-Derived Growth Factor-AB (PDGF-AB) quantification [130]. This effect can be attributed to CHS, as previously described [134].

An example of formulations intended for wound healing and containing CHS and HA blends is represented by the wound dressings based on chitosan hydrochloride (HCHS), 5-methyl-pyrrolidinone chitosan (MPC) and their blend with the anionic HA. They were prepared by freeze-drying and supplemented with the antimicrobial drug Chlorhexidine diacetate [135]. All dressings were characterized by mechanical resistance suitable for skin application. HA addition to CHS led to reduce the dressing hydration properties and to modulate drug release. Moreover, the dressing based on MPC showed the highest elastic properties and the best scavenger activity.

In another recent example of CHS polymer blends, CHSG/PEC/HA mini-capsules were prepared by inverse ionotropic gelation in presence of calcium chloride and subsequently freeze-dried to obtain a powder formulation for the delivery of MH bioactive components in the treatment of chronic skin ulcers [136]. Optimization of unloaded mini-capsules was performed using a DoE approach. The loading of MH fraction, rich in polar substances, into mini-capsules of optimized composition determined a significant increase in cell proliferation in comparison with the unloaded ones.

Eventually, films based on CHSG, poly(vinylalcohol) (PVA), poly (vinylpyrrolidone) (PVP) and SS were prepared by casting an aqueous dispersion containing a carvacrol (CVR)/clay hybrid (HYBD) for the delivery of CRV in infected skin ulcers treatment [137]. Different clays were investigated: montmorillonite (MMT), halloysite (HAL) and palygorskite (PHC). CRV incorporation in PHC reduced its volatility. HYBD showed 20% *w/w* CRV loading capacity and was able to preserve CRV antioxidant properties. Upon hydration, the optimized film obtained by a DoE approach, formed a viscoelastic gel able to protect the lesion area and to modulate CRV release. Chitosan derivatives were employed for the development of thermally sensitive hydrogels intended for the treatment of oral mucositis [138]. Trimethyl chitosan (TMC) and MPC were mixed with GP according to different polymer/GP molar ratios. The blends were characterized for gelation and mucoadhesive properties. The influence of molecular weight (MW) and TMC substitution degree (SD) on gelation temperature and time was investigated. The mixture characterized by the best properties, containing TMC with high MW and low SD mixed with GP in a 1:2 molar ratio, was loaded with benzydamine hydrochloride. The formulation was able to prolong drug release and to withstand the physiological mechanisms of removal. Moreover, the TMC/GP mixture showed antimicrobial properties also in absence of drug.

In addition to single unit medications, CHS-based nanosystems (nanoparticles, nanocomposites and nanofibers) have been recently developed for tissue reparation [123,139].

As long as CHS nanoparticles (CHS-NPs) are concerned, CHS ascorbate NPs, loaded with amoxicillin trihydrate, were developed for the treatment of atrophic vaginitis, which represents one of the most frequent complications of pelvic radiotherapy [140]. CHS ascorbate NPs were prepared by ionotropic gelation, using TPP as anionic cross-linker, and they were loaded into a freeze-dried polymeric matrix to facilitate vaginal administration. CHS ascorbate NPs showed mucoadhesion properties useful to enhance their permanence in the vaginal cavity and an inhibitory effect against two bacterial strains, *Enterococcus hirae* and *Streptococcus pyogenes*, higher than a CHS solution having the same polymer concentration. Based on these results, it was assumed that the nano-scale size and the high surface charge density of CHS ascorbate NPs more effectively support the interaction with the negatively charged surface of bacteria. Moreover, CHS ascorbate NPs were able to promote fibroblast proliferation and to enhance wound healing.

CHS-based nanocomposites (CHS-NCs) provide the dispersion of nanofillers (particle size ≤100 nm) into a CHS matrix. Several papers in the literature demonstrated that CHS-NCs, in the form of films, membranes, hydrogels and fibrous mats, are good candidates for wound healing, thanks to their enhanced properties, pertaining to both CHS and nanofillers, such as clay minerals and metallic nanoparticles. The wound healing potential of CHS-NCs based on the employment of clays was investigated by different research groups, and CHS-MMT has received particular attention in the field of biomedical applications. In particular, the loading of AgSD into CS-MMT NCs by an intercalation solution technique, in order to achieve a formulation intended for the topical treatment of chronic skin wounds, was investigated [141]. The effective intercalation of CHS chains in MMT interlayer spaces and the successful drug loading in the three-dimensional nano-structures were confirmed by means of solid-state analyses. Such systems were designed to combine AgSD antimicrobial activity with the wound healing properties of CHS and MMT and to reduce drug cytotoxicity towards fibroblasts and keratinocytes. The results demonstrated that AgSD loading into CHS-MMT NCs allowed exploiting AgSD antimicrobial effect, without delaying wound healing process. In a subsequent study, it was demonstrated the above-mentioned AgSD-loaded CHS-MMT NCs were able to protect human dermal fibroblasts from drug cytotoxic action, enabling their in vitro proliferation and stimulating their mobility (cell motility assay for wound healing) [142]. Moreover, AgSD loading into such nano-structures improved its bacteriostatic and bactericidal properties, particularly against *Pseudomonas aeruginosa* that is one of the main causes of wound healing impairment.

In an attempt to improve chitosan mechanical properties and water absorption capability, besides MMT, other clays have been investigated as reinforcing nanofillers to produce valuable CHS-NCs for wound healing. For instance, a powder consisting of NCs based on halloysite nanotubes (HTNs) and CHS oligosaccharide was set up [143], where association of HTNs with CHS was guaranteed by: (i) electrostatic interaction between positive charged amino groups of CHS and negative charges on the outer surface of HNTs and (ii) hydrogen bonding between amino and hydroxyl groups of CHS and Si-O residues of HNTs. CHS-HNTs powder proved to be biocompatible and able to enhance in vitro cell proliferation. Moreover, in vivo studies on a murine model confirmed the wound healing potential of CHS-HNTs nanocomposites: after 7 days treatment, the animals treated with the developed systems showed an early re-epithelialization and an advanced degree of hemostasis and angiogenesis in comparison with controls.

In the last decade, CHS-based nanofibers, obtained by the electrospinning technique, have been extensively proposed as valuable wound healing dressings, particularly due to their antibacterial and hemostatic properties combined to the enhanced properties promoted by electrospinning technique. However, to date, manufacturing electrospun nanofibers based on pure CHS is still challenging and electrospinning capability of CHS is known to greatly depend on its concentration, MW and deacetylation degree [144]. Chitosan-containing electrospun nanofibers could simultaneously act as wound dressings and drug delivery systems, and their antibacterial and wound healing properties could be improved by the addition of metallic nanoparticles [145], natural biopolymers [145] and/or therapeutic agents [146,147,148].

Since electrospinning of pure CHS solutions is challenging, CHS was also used as surface coating material for nanofibers made of PVA [149] or PCL/cellulose acetate [150]. It was demonstrated that surface coating with CHS improved the mechanical properties of alginate (ALG)/polyethylene oxide (PEO) electrospun fibers and slowed down their biodegradation in biological fluids. ALG/PEO fibers were crosslinked with calcium ions and then, they were coated with a CHS acetic aqueous solution: the partially protonated amino residues on CHS chains reacted with the carboxylate groups of ALG, resulting in the formation of a strong polyelectrolyte [151].

Nanofibrous scaffolds were successfully prepared by electrospinning polymeric blends containing CHS and pullulan (Pul) with glycosaminoglycans, HA or CS. Silver nanoparticles (AgNPs) were added to the polymer blends in order to prevent wound infections, thus enhancing cutaneous healing. A scaffold based on CHS and Pul and loaded with AgNPs was prepared for comparison [152]. TEM analysis demonstrated that all the electrospun scaffolds were based on nanofibers and that the presence of AgNPs did not modify their morphology. The nanofibrous AgNPs loaded scaffolds underwent enzymatic degradation induced by lysozyme and promoted fibroblast proliferation, without showing any toxic effect due to silver. Furthermore, the scaffolds demonstrated to maintain the AgNPs antimicrobial properties, despite silver was entrapped into the nanofibers. AgNPs CHS/chondroitin sulfate (CS) scaffold showed optimal fibroblast proliferation enhancement and antimicrobial properties, thus representing an interesting candidate for the treatment of chronic wounds.

Chitosan and Pul, associated with HA or CS, were further employed for the development of polymer-based scaffolds, prepared by electrospinning and insoluble in aqueous fluids, capable of mimicking the 3D extracellular matrix and consequently promoting wound healing [153]. The physical-chemical analyses demonstrated that the CHS/CS scaffold once hydrated shows more adaptability in terms of swelling and fiber roughening, thus conceivably allowing for optimal cell adhesion and migration. The same scaffold was indeed characterized by the best cell proliferation properties in vitro and the best healing properties in vivo in an animal model.

More recently, three types of polysaccharide-based scaffolds (CHS-based, CHS/CS-based, CHS/HA-based), intended as dermal substitutes for the treatment of infected wounds, were prepared by electrospinning with a simple one-step process. The scaffolds were loaded with norfloxacin as a free drug or loaded into MMT nanocomposite (hybrid-scaffolds) [154]. The scaffold containing CHS, and 1% norfloxacin loaded in the nanocomposite, demonstrated adequate stiffness to sustain fibroblast proliferation and antimicrobial properties suitable to prevent/treat non healing wound infection during the healing process.

Eventually, a novel composite nanosystem, consisting of CHS-coated Solid Lipid Nanoparticles (c-SLN) embedded in O-carboxymethyl chitosan (OCMCHS) and containing nanofibers, prepared by a two-step coating method was proposed as a potential tool for the local delivery of lipophilic anti-proliferative drugs in the local treatment of glioblastoma multiform, one of the most prevalent and aggressive brain tumors for which there is currently no therapy [155].

### 4.2. Alginic Acid

Alginic acid (ALG) is a linear polysaccharide consisting in repeated units of β-D-mannuronic acid and α-L-guluronic acid linked by α-1, 4 glycosidic linkages and derived from brown algae such as *Laminaria, Macrocystis* and *Ascophyllum sp*. [156].

It is generally recognized that alginates promote wound healing through two main mechanisms: (i) by maintaining wound bed moisture, which is functional to healing process by absorption of exudate excess. Chronic skin wounds are characterized by an excess of exudate that, besides producing maceration of surrounding tissue, is rich of matrix metalloproteinases (MMPs) and polymorphonuclear elastase that are tissue-destructive proteinase enzymes [122]; (ii) by activating wound macrophages in producing TNF-α, that has a direct role in the healing process [157,158].

Some researchers of the Department of Drug Sciences of Pavia University have designed ALG-based formulations intended for tissue repair. In particular, a powder formulation consisting of calcium alginate (CaALG) particles was prepared by freeze-drying beads obtained by ionic gelation method. The formulation was proposed for the combined delivery of PL and a model antibiotic drug, vancomycin hydrochloride (VCM), in chronic skin ulcers. The ALG-based particle formulations were able to load both VCM and PL without altering their activity and upon contact with a saline solution, they hydrate and modulate the release of VCM and of PGDF-AB, a growth factor representative of those contained in PL. Such formulation showed in vitro cell proliferation properties towards fibroblasts, and it was responsible for an increase in the number of cells in the proliferative phase [159].

In the last decade electrospun fibers characterized by nano- to micro-scale size have gained increasing interest as wound dressings. They combine the capability to act as both drug delivery systems and cell growth guidance. Since ALG can be electrospun only in association with spinnable polymers, its combination with dextran and PEO was investigated in order to evaluate the influence of polymer solution features (rheological properties, surface tension and conductivity) on electrospun fiber morphology by a DoE approach (full factorial design). The electrospun fibers were cross-linked with calcium ions or coated with poly(lactide-co-glycolide) and resulted to be biocompatible and able to support fibroblast proliferation, thus suitable to tissues regenerative purposes [160]. Moreover, the influence of PEO MW on ALG electrospinnability and on the mechanical properties of the nanofibers obtained was further investigated showing that mixing two PEO grades, at low and high MW, enhanced chain entanglement with ALG, functional to solution electrospinnability and improved the fiber mechanical properties [151].

Many papers have been published on the association of ALG with other polymers having wound healing properties in order to obtain a synergic effect. For example, the associations of CHS and fucoidan [161], SF [162], CHS and CL [163], N-carboxymethyl chitosan [164], have been investigated. Some researchers of the Department of Drug Sciences of Pavia University investigated the association of CaALG with HA, designing dressings composed by HA core-shell particles coated with CaALG and embedded in a VCM containing ALG matrix. The particles were loaded with PL, whereas the matrix contained VCM. They were characterized by optimal mechanical properties and were able to absorb a high amount of wound exudate, forming a protective gel layer on the lesion area. In vitro test on fibroblast cell line and ex vivo tests on human skin biopsy provided proof of concepts that the developed dressings were able to improve skin ulcers healing [165].

More recently, the same authors developed a dual-functioning platform with both neuroprotective and neuroregenerative potential to be used in the treatment of spinal cord injury (SCI). It consists of cross-linked ALG fibers containing the neuroprotective S1R agonist, 1-[3-(1,10-biphen)-4-yl] butylpiperidine (RC-33), incorporated in CHS acetate or glutamate films. The films were characterized by a controlled biodegradation rate and proved the formation of an interaction product between the anionic ALG chains and the cationic RC-33, responsible for a controlled RC-33 release in the physiological medium. The platform showed slower biodegradation and good compatibility towards human neuroblastoma cell line [166].

The same research group developed scaffolds based on biopolymers intended for restoring tissue integrity and for bacterial infection treatment in the periodontal pocket. The scaffolds consisted of electrospun nanofibers based on gelatin associated with low and high MW CHS and ALG. Physical-chemical (morphology, solid state, surface zeta potential and contact angle), and mechanical properties were investigated. The scaffolds were also characterized for their cytocompatibility, fibroblast and osteoblast adhesion and proliferation and antimicrobial properties [167].

Since ALG does not possess cell attachment sites or specific receptors and shows itself low cell adhesion properties [168], chemical modifications of ALG were proposed in the last decade to improve cell interaction and adhesion [168]. In particular, ALG hydrogels were modified with different peptides such as gelatin, collagen and arginine-glycine-aspartic acid (RGD) by the carbodiimide chemical procedure. Such modified alginates mimicked the ECM structure and were characterized by an improved cellular response.

### 4.3. Gellan

In recent years, a great variety of hydrogel-based systems for tissue repair has been studied. They consist of hydrophilic polymers and are characterized by a 3D network functional to tissue regeneration, providing a mechanical support and a guide for cell growth. In the last two decades, gellan (GG), a natural linear polysaccharide commercially produced by microbial fermentation of *Sphingomonas paucimobilis*, was proposed as a promising material in tissue engineering and regenerative medicine [169,170]. It consists of tetrasaccharide repeating units such as 1, 3-β-D-glucose, 1, 4-β-D-glucuronic acid, 1, 4-β-L-rhamnose, 1, 4-β-D-glucose, with one carboxyl side group. It is biocompatible, structurally similar to native glycosaminoglycans and possesses, upon hydration, viscoelastic properties near to those of common tissues [170]. Its use has been successfully proposed for intervertebral disc and cartilage repair [171,172,173,174,175].

Some authors have proposed the combination of GG with both inorganic materials, e.g., hydroxyapatite [176], inorganic clays [177], bioactive glass [178], calcium phosphate [179], demineralized bone powder [180,181] and other polymers, e.g., HA [182], SF [183], agar [184], lignocellulose [185], polyethylene glycol [186] to enhance its mechanical properties.

Similar to ALG, GG does not possess naturally cell adhesion properties, for this reason some authors functionalized GG chains with bioactive peptide or protein conjugates, in order to enhance cell adhesion and migration on GG scaffolds. Some authors modified GG with synthetic peptides derived from fibronectin (GRGDS) to enhance cell binding [187,188].

Some researchers of the Department of Drug Sciences of Pavia University developed an innovative in situ gelling system for local treatment of Inflammatory Bowel Disease (IBD). The system was composed by GG in association with methylcellulose and hydroxypropyl cellulose. The three polymers acted synergistically, increasing the permanence of the vehicle on the mucosa and forming a protective gel layer. In vitro tests performed on fibroblasts and Caco-2 cells confirmed system biocompatibility [189].

The same research group designed a GG-based composite system for the local delivery of RC-33, as a potential tool for the treatment of tissue nervous injuries [190]. The system consisted of cross-linked GG electrospun nanofibers embedded in a GG RC-33-loaded freeze-dried matrix and was intended to bridge the lesion gap, enhancing axonal regrowth. The formation of an insoluble interaction product between GG and RC-33 was responsible for a prolonged release of RC-33. Moreover, GG matrices were capable of absorbing a high buffer content, forming a gel with pronounced viscoelastic properties. At the same time, the presence of cross-linked nanofibers was responsible for an increase in matrix mechanical resistance.

### 4.4. Glycosaminoglycans

Glycosaminoglycans (GAGs) are unbranched polysaccharides constituted by repeating disaccharide units, composed of an hexuronic acid and an amino sugar residue, linked by glycosidic bonds. The variations in disaccharide composition distinguish the major classes of GAGs, such as HA, CS, dermatan sulfate, keratan sulfate, heparin and heparan sulfate. All GAGs are sulfated to different extents and characterized by molecular weights lower than 50 kDa, with the exception of HA, which is not sulfated and has molecular weight approximately ranging from 100 to 10.000 kDa. Moreover, the presence of ionizable groups (sulfates and carboxylates on hexuronic acids) is responsible for GAGs key properties [191].

Glycosaminoglycans are ECM components and play a vital role in binding cytokines and growth factors required for cell growth as well as a vast number of cell-surface receptors. They are highly polar and strongly interact with water molecules: this is fundamental to maintain osmotic pressure and hence provide mechanical support within tissues. Glycosaminoglycans capability to sequester proteins, such as growth factors and cytokines, enables regulation of their activity, either by acting as a co-factor or by limiting their bioavailability and consequently their degradation. In general, the biological activity and binding affinity of GAGs are related to their sulfation pattern, disaccharide unit sequence and 3D conformation; however, GAGs are also capable of unspecific binding of other positively charged proteins due to the negative charge provided by their numerous sulfate and carboxylic acid groups. Moreover, there are more specific interactions, not purely electrostatic, that involve hydrogen bonding, Van der Waals forces and hydrophobic interactions and depend on specific sequences and conformations of the GAG chains.

Glycosaminoglycans are dynamically synthesized and degraded by hydrolases and any sequestered proteins in the chain are released upon degradation.

Among GAGs, HA and CS have particularly attracted the attention and have been widely employed as key components in tissue engineering; the first research using GAGs in scaffolds for tissue repair appeared in the 1980s where HA was the first GAG investigated as a scaffold component. Hyaluronic acid is based on repeating disaccharide unit of D-glucuronic acid and N-acetyl-D-glucosamine attached by a β 1–3 bond, and the disaccharide units are joined by β 1–4 bond. It plays a role in ECM of several tissues and is involved in every step of wound healing since it has a fundamental role in promoting extracellular matrix secretion, reducing inflammation by inhibiting immune cell migration and maintaining homeostasis in healthy tissue. Moreover HA degradation increases tissue permeability and HA fragments further enhance angiogenesis and promote tissue healing processes [192]. Hyaluronic acid mediates receptor-driven detachment, mitosis and migration via interactions with CD44, RHAMM and ICAM-1 cell receptors. In its native form, it is a weak scaffolding material due to its rapid degradation caused in vivo by hyaluronidase and its high solubility. Moreover, it proved to be effective in remodeling phase, by tuning scarring [193]. Many examples of chemically modified HA derivatives are reported in the literature but they lead to new chemical entities whose safety and effectiveness profiles can be dramatically different from native HA.

Chondroitin sulfate is composed of D-Glucuronic acid and N-acetyl-D-galactosamine. It is generally highly sulfated with -SO_3_ groups occurring at C2 and/or C4 and/or C6 on galactosamine units, giving the following types: A: chondroitin-4-sulfate; C: chondroitin-6-sulfate; D: chondroitin-2,6-sulfate; E: chondroitin-4,6-sulfate. Similarly to HA, CS is a structural component of ECM able to induce cell differentiation is characterized by high solubility in aqueous fluids; differently from HA, CS promotes cell adhesion [194].

It has been associated to alginate to prepare 3D scaffolds by means of lyophilization. The structure was made of alginate chains gelled in presence of Ca^2+^ ions, causing the entrapment of CS chains in the ‘egg box’ structure of Ca alginate. The scaffolds had a 3D foam-like architecture with bubbles responsible for a high surface area and irregular texture whose 3D morphology, resembling ECM structure, creates a microenvironment suitable for cell adhesion and proliferation. [195]. The presence of CS allowed to stabilize platelet lysate (hemoderivative from platelets lysis rich in growth factors) demonstrating effective support to enhance fibroblasts and endothelial cells adhesion and proliferation on bubble surface.

Hyaluronic acid and CS have been compared to better evidence their peculiar properties on wound healing.

Hemostatic sponge-like dressings based on chitosan, in association with CS or HA have been designed and loaded with tranexamic acid (TA) to control bleeding in trauma and to improve skin tissue reparation [196]. Both CS and HA conceivably caused the occurrence of interactions with CHS and exerted a great influence on systems solubility hydration and morphology. When CHS was not associated with GAGs the dressings had a beehive structure with larger polyhedric cavities, interconnected by pores having oval or round shapes, whereas dressings based on CHS associated with GAGs were characterized by a markedly decreased cavity dimensions (200–300 μm) with smaller pore diameters (about 50 μm). This plays a key role in liquid absorption and ensured hemostasis, control of wound bed hydration, enhanced granulation phase and healing.

Glycosaminoglycans were proven to exert also a significant impact on dressing bioadhesion, which is fundamental to favor an intimate and prolonged contact between wound dressing and lesion, to avoid formulation detachment and increase hemostatic potential. In fact, the interaction of CHS with GAGs allowed consolidation of bioadhesive joint probably via hydrogen bonds. In vitro and ex vivo evaluations on fibroblasts and human skin, respectively, evidenced that the developed dressings enhanced cell proliferation. Glycosaminoglycans properties were not impaired by the presence of TA and this allowed to simultaneously control bleeding and healing in wounds.

Recently HA or CS were also associated to CHS and Pul in electrospun nanofibrous scaffolds [153]. In this type of formulations aqueous polymer blends based on polysaccharides having opposite charges, i.e., cationic CHS and anionic GAGs, were simultaneously electrospun and citric acid was added to the polymer blend, as cross-linking agent. The cross-linking process was activated by heating and made the scaffolds suitably resistant towards solubilization in aqueous fluids: this behavior seems attributable to a felting phenomenon occurring when water is released from the electrospun scaffold, resulting in local physical multi-entanglement between fibers, that could not be released by simple hydration. Chondroitin sulfate proved to be a crucial component and allowed the best performance in skin healing in vivo (murine burn/excisional model) and correspondingly, it determined the best proliferation properties of fibroblasts and endothelial cells in vitro. The better performance with respect to HA was attributed to a greater adaptability in terms of swelling and fiber roughening once hydrated, thus allowing optimal cell adhesion and migration, conceivably due to CS protrusion/release from the fibers. The presence of GAGs together with CHS in composite scaffolds allowed to tune system degradation. The nanofibrous structure of scaffold without GAGs was completely lost after 10 days contact with lysozyme, which is normally secreted by macrophages and polymorphonuclear neutrophils during the inflammatory phase of the healing process. The presence of CS and mainly of HA in the scaffolds slowed down the degradation process and determined higher resistance towards degradation, conceivably due to the interaction of chitosan aminogroups (positively charged) with either sulfate groups of CS or carboxylic groups of HA (both negatively charged). Such an interaction could prevent loss of system morphology, although CHS enzymatic degradation occurred [152].

Clay minerals, either MMT or HNTs, have been loaded in CS scaffolds [197]. MMT inclusion in the polymeric matrix of the scaffolds caused interlayer space enlargement, causing the biopolymer intercalation into its galleries, resulting in a deep interaction between the scaffolds matrix and clay mineral. HNTs or MMT in CS scaffolds were able to sustain homogeneous fibroblast spreading all over the scaffolds and their growth up to confluence, maintaining a cell fusiform structure and aligned and elongated cytoskeleton filaments. Their capability to enable cell attachment and adhesion, with negligible proinflammatory activity, was probably due to their morphological 3D structure-assisted cell homing, and this could further facilitate wound healing in vivo.

## 5. Biodegradable Aliphatic Polyesters

Aliphatic polyesters are representatives of synthetic biodegradable polymers. The most commonly used for tissue repair and regeneration are polylactide (PLA), polyglycolide (PGA), poly(ε-caprolactone) (PCL), their copolymers and derivatives by PEG or other polymers and materials [198,199,200,201]. These polymers have been widely studied for decades in drug delivery applications such as microparticles, NPs, implants and several pharmaceutical products are on the market [202,203]. Due to their consolidated use in the pharmaceutical field, PLA, PGA and PCL biodegradability and biocompatibility is well known, and the polymers are approved by the regulatory agencies American FDA and European Medicine Agency (EMA) in injectable drug products. These reasons led them to be also widely studied for biomedical applications in tissue repair and regeneration. Recently, an interesting and complete paper by Ramot et al. updated the concept of biocompatibility applied to PLA and its copolymers [204]. The authors confirmed the polymers can generally be considered safe and they do not elicit genotoxic response. However, an inflammatory response can be highlighted after implantation, depending on the site of implant and its size. Moreover, the authors advise caution when these polymers are changed by chemical means or by drug incorporation, as they can change their original properties and degradation rate, which can result in a change in the inflammatory reaction. The paper underlines how reaction to implant of PLA and its copolymer is always local and close to the implanted material and not systemic in nature [204].

### 5.1. Synthesis and Properties

**Figure 2:**
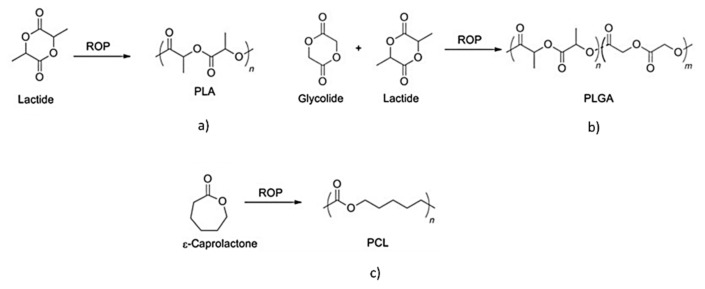
Common synthetic pathways of: (**a**) polylactic acid (PLA), (**b**) polylactide-co-glycolide (PLGA), (**c**) poly(ε-caprolactone) (PCL), by ring-opening polymerization.

Both PLA, PGA, PCL and their copolymers are synthetic polymers and can be obtained by a condensation reaction or by ring-opening polymerization (ROP), as schematized in Figure 2.

Polylactic acid comes from the chiral lactide molecule, thus four different types of polylactic acid are available: poly(l-lactic acid (PLLA), poly(d-lactic acid) (PDLA), poly(dl-lactic acid) (PDLLA) and mesopolylactic acid. Specifically, lactide L isomer gives the semi-crystalline form of polymer with a glass transition temperature (Tg) of 55–60 °C. D isomer has an amorphous structure and PDLLA is a semicrystalline polymer whose Tg is about 60 °C, similar to that of PLLA. Glass transition temperature influences the solid state behavior of polymers and their mechanical properties. Since the latter are of utmost importance when developing scaffolds for tissue regeneration, this topic has been thoroughly investigated in the literature. Shao and colleagues [205] investigated the crystallization behavior of PLLA/PDLA and reported that formation of stereocomplex crystallites (SC) improved the mechanical properties of PLLA/PDLA compared to polymer blends homochiral crystallites (HC). The behavior is explained by a more compact structure in SC crystallites leading to higher melting temperature of the polymer blend, higher heat deflection temperature and better hydrolysis resistance [205].

The molecular structure of PLA makes it quite hydrophobic, thus its hydrolysis rate is slower than that of PGA and it is more useful as material to manufacture scaffolds for tissue regeneration purposes. PLA complete resorption can take even 12 months after implantation in the human body. However, PLA degradation rate depends also on its MW, crystalline or amorphous state, and addition of excipients or combination with other polymers materials [206].

Polyglycolide is a highly crystalline polymer with melting point greater than 200 °C and glass transition temperature around 35–40 °C. It was used to develop the first biodegradable synthetic suture, DEXON, in 1970, but is not used alone in manufacturing scaffolds for tissue regeneration because of the following drawbacks: (i) while polyortoesters are soluble in common organic solvents, PGA is soluble in hexafluoroisopropanol (HFIP) that is a highly toxic solvent; (ii) PGA has high degradation rate that results in instability [207]. Polyglycolide degrades through backbone hydrolysis mechanism, and its hydrophilicity results in fast hydrolysis rate. This property represents a drawback when the polymer is proposed for tissue engineering purposes because it causes scaffold instability with mechanical failure. Moreover, the high amounts of glycolic acid, generated by PGA hydrolysis, can be responsible for a strong, undesired inflammatory response [207]. 

Poly(ε-caprolactone) is a semicrystalline polyester with a melting temperature of about 55–60 °C and a glass transition temperature at −60 °C. Therefore, this polymer is in rubbery state when administered in the human body and is highly permeable. It is of great interest because PCL can be obtained from cheap starting material and has high solubility in common organic solvents such as chloroform, dichloromethane, carbon tetrachloride, benzene, toluene, cyclohexanone and 2-nitropropane at room temperature, low melting point and an exceptional ability to form blends with a variety of polymers with plasticizer effect [208].

Poly(ε-caprolactone) has very low hydrolysis rate due to its high degree of crystallinity. For this reason, and also due to its low glass transition temperature (below zero) that makes it rubbery at room temperature, PCL is commonly blended with other polyesters and polyethers, and PCL-based copolymers are obtained by copolymerizing ε-caprolactone with other cyclic esters with the main purpose of accelerating its hydrolysis [209]. The rubbery PCL has been frequently copolymerized with glassy PLA, which has higher hydrolytic degradability, as an effective strategy to obtain a Polylactide-co-polycaprolactone (PLA-PCL) copolymer whose higher rate of degradation can be modulate according to the copolymer composition.

Block copolymers made from PLA and PGA (Polylactide-co-glycolide (PLGA)) or PLA and PCL with different homopolymer ratios can usefully improve or modulate homopolymer properties, in order to obtain materials with characteristics suitable for the addressed purpose.

Summarizing, the polyorthoesters main degradation mechanism is hydrolysis of ester functions present in the (co)polymer backbone, formation of non-toxic oligomers and ultimately monomers (i.e., lactic acid in case of PLA) which are eliminated through the human metabolic pathways, and excreted mainly, after multiple transformations, through the lungs as water and CO_2_. Their degradation time varies according to polymers molecular weight and structure. Non-specific esterase and carboxypeptidases can also be partially involved in their biodegradation.

Due to the importance of these polymers in the pharmaceutical and biomedical area, the degradation mechanism of PLA, PLGA, PCL and their copolymers and blends has been widely investigated in the past years [210,211,212,213]. The generally accepted degradation mechanism of aliphatic polyesters, in aqueous media, is hydrolysis via random cleavage of the ester bond, which is mainly controlled by: amount of absorbed water, diffusion coefficient of chain fragments within the polymer and of degradation products solubility [214]. The hydrolytic degradation happens through two different mechanisms: (i) surface or heterogeneous reactions and (ii) bulk or homogeneous erosion. Prevalence of one mechanism depends on polymer hydrophilicity and crystallinity [215,216]. The biodegradation rate of PLA, PLGA, PCL, their copolymers and derivatives, ranges from 2 months up to 12 months, depending on polymer composition. This makes them suitable for application in tissue regeneration by scaffolds made from these polymers which should work supporting tissue regrowth. The advantage of biodegradable materials is that they biodegrade after implantation in the human body and their degradation products are metabolized and excreted by the human body. Therefore, the implanted polymer scaffold neither needs to be removed whenever the tissue has been restored nor causes adverse reaction due to its degradation products., In these terms is envisaged that biomaterial degradation rate should be synchronized with tissue regrowth rate.

Mechanical properties of PLA, PLGA, PCL, their copolymers and derivatives need to be identified when designing a scaffold for tissue regeneration in order to match those of tissue to be restored. These polymers have very versatile mechanical properties, depending on their composition and on scaffold morphology. Both crystallinity and polymer MW affect the polyesters mechanical properties in such a way the high crystallinity and high MW demonstrate higher mechanical properties. PLA is a brittle polymer whose mechanical strength (in terms of Young modulus values) can range between 3–5 kPa to 2–4 MPa [206,217]. Polylactide elasticity increases, and can be tuned, when blended or copolymerized with PCL [217].

Moreover, since scaffolds intended for implantation in the human body, as well as injectable pharmaceutical products based on PLA and copolymers, should be sterile, also sterilization processes and their influence on the polymers degradation have been investigated. Ionizing irradiations (either gamma or beta irradiation) and ethylene oxide are the sterilization methods that can be applied to aliphatic polyesters because the polymers do not stand sterilization either by steam or dry heat. Results reported in the literature outline that sterilization either by gamma-irradiation or electron beam, promote polymers degradation by hydrolytic scission with fasting polymer molecular weight decrease. The polymers degradation induced by gamma-irradiation is higher in copolymers such as pegylated PDLA. Moreover, oxygen molecules which permeate the polymer matrix, and free radicals formed by irradiation treatments can promote changes in polymer structure, which continue with time after irradiation [218,219]. Ethylene oxide (ETO) sterilization seems to be less detrimental to PLA and copolymers, as underlined by Jain and colleagues [220]. However, it should keep in mind that ETO is toxic and after sterilization with ETO, the product should go through a quarantine storage time in order to desorb the gas [218,219,220,221,222].

Some of properties of these polymers relevant to tissue regeneration purposes are summarized in Table 3.

### 5.2. Examples of PLA and Derivatives Scaffolds for Tissue Regeneration

Due to the aforementioned properties and versatility, PLA, PGA, PCL, their derivatives and copolymers have been studied with different tissue regeneration purposes such as: bone, blood vessels, esophagus, trachea.

In the following paragraphs some examples of experimental works published by Italian research groups on soft tissues regeneration are reported. Please note that the following examples do not want to be exhaustive of the wide literature on the topic.

Even if exceeding the aim of this review, it is worth to highlight these polymers have been extensively studied for bone regeneration purposes, due to their suitable mechanical properties and the ability to modulate them depending to their blending and copolymerization [201,206,225,226,227,228]. Bone tissue can undergo serious degenerative problems, i.e., due to accidental nonunion fractures or pathologies such as bone tumors. Mechanical properties are a main challenge when thinking to scaffold for bone substitution that should stand high pressures. Moreover, biodegradable scaffolds made from PLA, PGA, PCL, their blends and derivatives have the advantage they resorb and do not need a second surgery to be removed after implantation.

As long as soft tissue is concerned, esophagus is a peculiar example of hollow, fibromuscular tube. Congenital esophageal malformations such as atresia, and acquired such as chronic gastroesophageal reflux, Barrett’s esophagus, malignant esophageal cancer, and strictures often require surgical intervention, such as esophagectomy and endoscopic mucosal resection, and consequent replacement is mandatory to maintain gastrointestinal continuity and functionality. The resected tract must be replaced with autologous conduit, but this procedure is associated with significant morbidity, rupture of suture or stenosis and a mortality rate of about 4%. Moreover, esophagectomy is a very invasive surgery that could induce an intense systemic inflammatory response (SIR) and postoperative pneumonia infection. The limitations of current surgical procedures highlight an unmet need for tissue engineered grafts for esophageal reconstruction. Recently, PLA-PCL copolymer has been thoroughly investigated by R. Dorati et al. for esophageal regeneration, different polymer blends and cutting-edge manufacturing techniques have been evaluated. A polymer patch was designed combining synthetic Polylactide—polycaprolactone copolymers (PLA-PCL) 85:15 (181,492 Da Mw), and chitosan (110,000 Da MW) biopolymers, tailoring patch properties to esophageal tissue characteristics. Stable multilayered patches (1L (1 layer), 2L (2 layers), and 3L (3 layers)) were obtained by temperature induced precipitation method and in vitro investigation of the functional patch properties in simulated physiologic and pathologic conditions demonstrated that the chitosan layer (patch 3L) decreased patch stability and cell adhesion, while improved cell proliferation. Patches 2L and 3L complied with physiological esophageal pressure (3–5 kPa) and elongation (20%) [217]. The same authors thoroughly investigated PLA-PCL/CHS blends by electrospinning, obtaining electrospun matrices nanofibers in the range size 800 nm. Chitosan was loaded into the nanofibers up to 27.2% (*w/w*) without modifying nanofiber shape, it was stabilized by TPP crosslinking and only 6% of the loaded chitosan resulted to be on the nanofiber surface. Blending PLA/PCL with chitosan polymer resulted a useful strategy to improve wettability performances of the electrospun matrices together with cell attachment and proliferation. The presence of chitosan in the nanofibers accelerated the electrospun membranes degradation in vitro [229]. Furthermore PLA/PCL blends with different ratios (namely 85:15 and 70:30) were investigated manufacturing bilayer electrospun matrices that were engineered with porcine bone marrow mesenchymal stem cells (p-MSCs). The authors set up a reproducible and effective method to cellularize the electrospun matrices. Furthermore, the investigation provided interesting information on patches stability in the different in vitro experimental conditions tested [230]. Eventually, electrospinning process parameters and their influences on physical-chemical and biological properties of PLA/PCL matrices were investigated showing that electrospinning process parameters significantly affect the electrospun fiber orientation. Namely, greatest fiber orientation was achieved when all the three input parameters (voltage, flow rate and mandrel rotation speed) were at their maximum value. Three different cell lines, fibroblast NIH 3T3, Neuro 2α (N2α) and murine mesenchymal stem cells (mMSC) were tested on electrospun fibers. N2α cells resulted to be more challenging in terms of adhesion to electrospun substrates, with as low as 45% vitality after 3 days incubation. However, the cells significantly proliferate in the following days of incubation until day 6 reaching about 90% cell viability. Moreover, N2α and mMSC cell growth demonstrate electrospun matrices prepared with 25 G needle and rotating mandrel to be a preferential substrate for cell proliferation [231,232].

An interesting in vitro study was conducted by Pisani et al. [233] evaluating the reaction of monocytes and macrophages cells after contact with a biomaterial. The results give a preliminary scenario regarding immune system reaction after contact with electrospun matrices made from PLA-PCL 70:30 ratio, which can be useful since regenerative process has to consider the role of immune system after surgical implantation of a polymer-based matrix. The authors evaluated the in vitro acute response induced by PLA-PCL electrospun matrices, after 3 days contact with naïve macrophages (M0) and their ability to modulate M0 polarization into M1 (pro-inflammatory) and/or M2 (anti-inflammatory) macrophage phenotypes. Biological characterization included MTT ((3-(4,5-dimethylthiazol-2-yl)-2,5-diphenyltetrazolium bromide), LDH (Lactate dehydrogenase activity) and Live/Dead assays, immunological characterization by ELISA for cytokine-expression levels determination. The results showed an initial pro-inflammatory response (after 24 h) characterized by release of Tumor Necrosis Factor (TNF)-alfa, interleukin (IL)-6 and IL-8 cytokines which decreases and is substituted by preferential anti-inflammatory response rising after 72 h, with IL-10 release and elongation of macrophages typical of M2 cell polarization. These results confirm PLA-PCL electrospun matrices can be eligible as support to enhance tissue regeneration promoting an anti-inflammatory response [233].

The encouraging positive results achieved in the mentioned experimental works [231,232,233] led to start an in vivo experimentation on animal model involving pig esophagus transplant that is currently ongoing (data not published yet).

Here below some examples of experimental works involving polyesters combinations and new derivatives for tissue regeneration purposes, are reported.

PLA–PCL composite mats were successfully loaded with graphene nanoplatelets (GNPs), at 0.5 wt % to 4 wt % concentration, by electrospinning technique [234]. The set-up electrospinning process confirmed reliable in promoting parallel orientation of GNPs’ base plane to the jet flow direction with a strong binding among the two-dimensional (2D) graphene sheets and the thermoplastic polymer matrix and thus impacting the thermal behavior of the composite electrospun matrices with its significant enhancement. GNPs enriched PLA–PCL electrospun mats loaded with the highest amount of GNPs (4%) exhibited thermal conductivity of 1.27 ± 0.008 W/m K and thermal diffusivity of 1.07 ± 0.068 mm^2^/s. As far as mechanical properties, samples containing the lowest GNPs loadings (GNPs_0.5–1%) showed the highest peak stress values (around 6.6 ± 1.3 MPa) and achieved the maximum elastic modulus values of 36.3 ± 7.6 MPa and 33.6 ± 5.9 MPa, respectively.

At the same time, GNPs were also liable of slowing down PLA-PCL degradation rate in simulated physiological environment where no toxic impurities and degradation products were pointed out up to 60 d incubation. Furthermore, preliminary biologic tests proved the ability of the matrices to enhance fibroblast cells attachment and proliferation probably due to their unique 3D-interconnected structure.

Another example is a PLA derivative with poly(1,5 cyclooctadiene-co-5-norbornene-2-methanol). The PLA derivative composed by 95% mol of D,L lactide and, 5% mol of poly(1,5 cyclooctadiene-co-5-norbornene-2-methanol (named LP-3055) was synthesized with the rationale to improve the toughness and tensile properties of the related PLA homopolymer that could overcome PLA homopolymer brittle nature. The approach consisted in addition of rubbery domains into the backbone of PLA homopolymer. The rubbery phase makes available supplementary dissipation energy during the deformation process, resulting in an increase of block copolymer toughness [235,236]. Dorati and colleagues investigation demonstrated LMP-3055 was neither cytotoxic nor it released toxic leachable substances during incubation in cell culture medium Dulbecco’s Modified Eagle’s Medium (DMEM), before and after sterilization by gamma irradiation at 25 kGy, keeping cell biofunctionality [223]. 3D scaffold and 2D film made fromLMP-3055 were prepared and tested with positive results: SEM analysis revealed the porous structure of 3D-2 and 3D-3 scaffolds with well interconnected pores; in vitro degradation of 3D scaffold polymer matrix (evaluated as MW variation) and water uptake exceeded those of pristine polymer rand 2D film; mass loss was controlled, resulting in good stability of the construct and ability to maintain its physical integrity. Compressive and tensile properties resulted to be related to the structural features of scaffolds. Indeed, the trabecular structure of 3D scaffold led to a system with more limited tensile strength with respect to the 2D film. The authors concluded that LM-3055 can be a candidate for different tissue engineering applications, when a tough polymer is required [223].

## 6. Regulatory Aspects

To talk about the regulatory aspects of the employment of biomaterials, either bio-inert or bioactive, in products intended for tissue repair and regeneration, firstly is needed to understand what regenerative medicine means. Regenerative medicine is not a legally binding definition and covers any multidisciplinary procedure used in pathological/aesthetic situations with the aim to heal impaired functions, to replace or repair malfunctioning or damaged tissues, and to stimulate the body’s intrinsic capacities for regeneration. The term tissue-engineered products or medicines (TEP) instead, together with gene therapy and somatic cell therapy medicines are categorized as Advanced Therapy Medicines (ATM) according to Regulation (EC) 1394/2007. Tissue engineered medicines may contain engineered cells of human or animal origin, living or not living tissues or additional substances such as cellular products (DNA, genes), biomolecules, biomaterials, chemical substances, supports or matrices. Specifically, TEP contain cells or tissues modified with the aim to repair, regenerate or replace human tissue. They express activity of regeneration, repair or replacement of cells and tissues by a pharmacologic, metabolic or immunologic mechanism of action.

Representative examples are: fibroblasts for reconstructing the skin in burned patients (expansion), stem cells from bone marrow after heart attack (reconstruction of the damaged cardiac tissue), musculoskeletal cells to replace the urethral sphincter (muscular tissue) in incontinence, and so on. A special family of ATMs are the so-called Combined advanced therapy medicinal products (CATMPs) that contain one or more medical devices as an integral part of the medicine. An example are cells embedded in a biodegradable matrix or scaffold. The classification of ATM, in particular the definition of so-called borderline products between the various therapies is still at the stage of Reflection paper (EMA/CAT/600280/2010 rev.1). Particularly a clear cut off between an ATMP and medicinal product does not exist, and the applicant is required to ask for advice in order to obtain a classification and to plan for an appropriate development strategy. Products containing or consisting exclusively of non-viable human or animal cells and/or tissues, and which do not act principally by pharmacological, immunological or metabolic action, are out of the scope of the above definitions of ATM. In the author’s understanding, some of the products described in the present review belong to the category of Advanced Therapy products, either combined or not combined, wherein biomaterials serve as additional substances or function as scaffold or matrix. The consequence of this is that they are medicinal products to all effects. This must be clear since the very beginning of lab development phases especially within the prospective of filing a patent or conceding a utilization license. Some others of the described products may belong to the categories of Medical Devices or Cosmetics. If this is the case, the approach to regulatory issues is different depending on the intended use. Particularly, use of biopolymers in a medical device automatically place the product in the category of medical devices containing substances, which are subject to a special scrutiny on behalf of the regulatory bodies. The declared mechanism of action of such products should be physical or mechanical, precluding any pharmacologic, metabolic or immunologic mechanisms of action.

A few examples of likely product classification are taken from the review chapters.

Silk fibroin, proposed in films, scaffolds, mats and fibers made of either in regenerated or native form, offers an example of the versatility of biomaterials. It is possible to use silk fibroin both in medical devices and in advanced therapy products. Silk Fibroin film scaffolds proved capable of significantly improving wound healing with respect to commercial dressings in a clinical trial paving the way for a clinical use of SF films for skin regeneration [52]. Such a product is likely to become a medical device provided the mechanism of action of SF is clarified. Instead, the use of RSF scaffolds for infrapatellar delivery of Adipose Stromal Vascular Fraction as Feeder-Layer for Cartilage regeneration is eligible as an Advanced Therapy [51]. Similarly, use of SF mats loaded with mesenchymal stem cells with the aim of eliciting subcutaneous compartment’s vascularization for nutrient supply of cells involved in skin regeneration process is likely to become an Advanced Therapy product [55,56]. In both cases it should be decided if they are considered combined ATMs or not. This will depend on the polymeric scaffold behavior. As anticipated above a clear distinction between the two is not available yet in the regulatory scenario.

Polylactide and polycaprolactone represent another class of polymers which have well-established regulatory status. Being biodegradable and biocompatible, they have been studied for about 50 years for application in the medical and pharmaceutical field, also with different tissue regeneration purposes such as bone, blood vessel, esophagus and trachea. The polymers are approved by the main regulatory agencies FDA and EMA for use in the human body and biodegradable scaffolds made from PLA and/or PCL have the advantage that they resorb and do not need a second surgery for removal after implantation. In example, in case of bone fractures or bone resection caused by tumors, scaffolds promoting tissue regeneration can be a useful support to the natural self-organizing bone regeneration. Such one product obtained through coating a decellularized and deproteinized bone matrix of bovine origin with a special grade of PLA, has been studied [223] and could be eligible as a medical device. Instead, PLA-PCL electrospun matrices proved capable to induce modifications in macrophages (M0) by modulating in vitro M1 and/or M2 macrophages polarization, thus promoting an anti-inflammatory response [233]. Based on the type of cell loading and on the mechanism of action such matrices could then be eligible to become an advanced therapy product.

Other biopolymers of natural origin of polysaccharidic nature such as ALG, HA, CS, traditionally employed in medical dressings as well as additional substances in medicines, are presently under scrutiny since they are considered borderline products. This means that, depending on the product intended use, the producer needs to clarified which is the product principal mechanism of action and, also depending on the administration route, it might be required to extensively scrutinize their in vivo fate in order to classify the final product as medical device or drug product. 

Eventually, special regulatory concerns are raised by CHS and CHS derivatives, due to their animal origin and multifaceted biological activity which make them very attractive, but at the same time makes it difficult to exclude a pharmacological or immunological mechanism of action. As a result of this, they are not immediately accepted as polymeric excipients.

## 7. Future Perspectives 

All the discussed biomaterials are promising in the field of tissue regeneration. Generally speaking, recent trends are towards tissue engineered scaffolds, i.e., hybrid systems combining cells with polymer materials with particular focus on tissue restoring ability of mesenchymal stem cells (MSC). Moreover, different biologic molecules such as growth factors and cytokines can usefully improve regeneration activity.

As long as silk and silk derivatives is concerned, during the last two decades, both in vitro and in vivo results underlined the high therapeutic potential of SF and SS based materials for tissue engineering applications. Despite the intense interest of the scientific community, the clinical applications of these two proteins are still far.

The routinely clinical use of silk protein-based scaffolds was strictly correlated to their large-scale production, maintaining both high-quality level and batch-to-batch consistency. All production steps need to be performed according to the Good Manufacturing Practices and overcoming some problems such as low production yield, high costs and lack of infrastructure and expertise. Silk proteins were produced by living organisms and not synthesized in a laboratory; this aspect complicates the validation of all production steps. A full defined characterization of raw materials and final products must be conducted to obtain reproducible, safe and effective constructs for tissue engineering applications.

As long as PLA, PLGA and PCL are concerned future trends are towards to derivatize these polymers in order to modulate their properties (such as the mechanical ones) depending on their applications.

## 8. Conclusions

The review highlights how wide is the area of biomaterials for application in tissue engineering, and which tremendous impact biomaterials are having and will have for future clinical applications. Continued growth of this field depends both on the development of new materials, improved scaffold processing techniques and improved cell manipulation techniques. The three factors are interdependent and should be optimized in order to further improve tissue regeneration opportunities. Despite the wide research carried on tissue regenerative approaches and biomaterials, only few products reached clinical market. The gap is due to different reasons such as poor identification of clinical critical adoption criteria, lack of translation from early research process and its clinical application, fail of clinical trials, lack of compliance to regulatory constraints. Hopefully, this gap will be reduced in the near future, due to optimized research in cell therapy combined to biopolymers.

## Figures and Tables

**Table 1:** Principal properties of silk fibroin and silk sericin in systems for reparation and regeneration. Mechanical properties are referred to ultimate tensile strength.

Biomaterial	Scaffold Type	Biocompatibility	Biodegradability	Sterilizability	Mechanical Properties	Porosity-Pore Size
Silk fibroin	Sponges	Good	Months-Years [14]	Autoclave/Irradiation	5–100 kPa [15]	100–1000 µm [16]
	Hydrogels	Good	Days-Months [14]	Filtration 0.22 µm/Irradiation	20–90 MPa [17]	10–350 µm [18,19,20]
	Films	Good	Days-Months [14]	Filtration 0.22 µm/Irradiation	10–100 MPa [21]	Not reported
	Mats and fibers	Good	Months-Years [14]	Autoclave/Irradiation	2–18 MPa [22,23]	0.5–12 µm [23,24]
	3D Printed scaffold	Good	Not reported	Filtration 0.22 µm/Irradiation	Modulable [25]	Modulable [25]
Silk sericin	Sponges	Good	Hours-days [26]	Filtration 0.22 µm/Irradiation	0.2–1 kPa [27]	Not reported
	Hydrogels	Good	Hours-days [28]	Filtration 0.22 µm/Irradiation	0.6–6 kPa [27]	20–300 µm [28]
	Films	Good	Hours-days [29]	Filtration 0.22 µm/Irradiation	10–40 MPa [30]	Not reported

**Table 2:** Principal properties of collagen in systems for reparation and regeneration.

Scaffold Types	Biocompatibility	Biodegradability	Sterilizability	Mechanical Properties (E Modulus)	Porosity
Hydrogels [80,81,89,91] Fibers [90,97,99,100,106] Films [83,92] Hybrids [82,93,94,95,96,103,107] Sponges [79]	Good as component of ECM matrix [75,80] Possible concerns of immunogenic effects [79,80]	Hours/days if not crosslinked [97,99] Modulable up to 6–24 months after crosslinking [80,84,85,87,89]	Gamma irradiation or ethylene oxide [80,108]	Generally poor without crosslinking For hydrogels, up to kPa. For fibers and films up to MPa after chemical crosslinking [80,84,85] Modulable by grafting/mixing with polymers [97,99,104]	Tuning by cross linking [85] and by nanofiber engineering [86]

**Table 3:** Principal properties of polyesters in systems for reparation and regeneration.

Biomaterial	Scaffold Type	Biocompatibility	Biodegradability	Sterilizability	Mechanical Properties (Young’s Modulus)	Porosity-Pore Size
Polylactic acid	Fibers (nano and micro)	Good [204] Approved by FDA and EMA for human use in injectable drug products	Months, depending on its Mw [210,214]	Ionizing radiations (gamma and beta), Ethylene oxide (ETO) [216,219,220]	3–5 kPa [217]	1–100 µm [217]
	Films					
	3D scaffolds				2–4 MPa [206]	10–900 µm [223]
Polyglycolide (due to its inherent hydrolitic instability it is used in blends or copolymerized with PLA)	Fibers (nano and micro)	Good [204] Approved by FDA and EMA for human use in injectable drug products	Weeks–months depending on Mw [207]		Can be tuned depending on its blending or copolymerization with PLA	1–100 µm [217]
	Films					1–100 µm [217]
	3D scaffolds					10–900 µm [223]
Poli(ε-caprolactone) (Mostly used as PLA-PCL copolymer or in blend with PLA)	Fibers (nano and micro)	Good Approved by FDA and EMA for human use in injectable drug products	Months–Years depending on Mw and on derivative [213,224]		3–5 kPa [217] Can be tuned depending on its blending or copolymerization with PLA	1–100 µm [217]
	Films					

## Data Availability

Not applicable.

## Abbreviations

1L	1 layer
2D	two-dimensional
2L	2 layers
3D	three-dimensional
3L	3 layers
AgNPs	Silver nanoparticles
AgSD	silver sulfadiazine
ALG	alginate
ATM	Advanced Therapy Medicines
CaALG	calcium alginate
CAD	computer Aided Design
CATMPs	Combined advanced therapy medicinal products
CHS	Chitosan
CHSG	chitosan glutamate
CL	Collagen
CS	chondroitin sulfate
c-SLN	coated Solid Lipid Nanoparticles
CVR	carvacrol
DHT	dehydrothermal treatment
DMEM	Dulbecco’s Modified Eagle’s Medium
DoE	Design of Experiment
ECM	extracellular matrix
EDC	Ethyl-3-(3-dimethylaminopropyl)carbodiimide)-based
EMA	European Medicine Agency
ETO:	Ethylene oxide
FDA	Food and Drug Administration
GAG	glycosaminoglycans
GFs	growth factors
GG	gellan
GLY	glycine
GNPs	graphene nanoplatelets
GP	glycerophosphate
HA	hyaluronic acid
HAL	halloysite
HC	homochiral crystallites
H-chain	heavy chain
HCHS	chitosan hydrochloride
HTNs	halloysite nanotubes
HYBD	clay hybrid
IBD	Inflammatory Bowel Disease
IL	interleukin
L-chain	light chain
M0	naive macrophages
M1	pro-inflammatory macrophages
M2	anti-inflammatory macrophages
Mats	matrices
MBG	mesoporous bioactive glasses
MH	Manuka Honey
MMPS	metalloproteinases
mMSC	murine mesenchymal stem cells
MMT	montmorillonite
MPC	5-methyl-pyrrolidinone chitosan
MSC	mesenchymal stem cells
MW	molecular weight
NCs	nanocomposites
NPs	nanoparticles
OCMCHS	O-carboxymethyl chitosan
PCL	poly(ε-caprolactone)
PDGF-AB	Platelet-Derived Growth Factor-AB
PDLA	poly(d-lactic acid)
PDLLA	poly(dl-lactic acid)
PEC	pectin
PEO	polyethylene oxide
PGA	polyglycolide
PHC	palygorskite
pI	protein isoelectric point
PL	platelet lysate
PLA	polylactide
PLA-PCL	Polylactide-co-polycaprolactone
PLGA	Polylactide-co-glycolide
PLLA	poly(l-lactic acid)
p-MSCs	bone marrow mesenchymal stem cells
PRP	platelet rich plasma
Pul	pullulan
PUR	polyurethane
PVA	poly(vinylalcohol)
PVP	poly (vinylpyrrolidone)
pxch	chemically effective crosslinking
pxel	elastically effective crosslinking
RC-33	1-[3-(1,10-biphen)-4-yl] butylpiperidine
RGD	Arg-Gly-Asp
ROP	ring-opening polymerization
RSF	regenerated silk fibroin
SC	stereocomplex crystallites
SD	substitution degree
SF	silk fibroin
SS	sericin
TA	tranexamic acid
TEP	tissue-engineered products or medicines
TMC	Trimethyl chitosan
TNF	Tumor Necrosis Factor
VCM	vancomycin hydrochloride
